# The role of HDAC3 and its inhibitors in regulation of oxidative stress and chronic diseases

**DOI:** 10.1038/s41420-023-01399-w

**Published:** 2023-04-18

**Authors:** Ruyuan He, Bohao Liu, Boxin Geng, Ning Li, Qing Geng

**Affiliations:** 1 Department of Thoracic Surgery, Renmin Hospital of Wuhan University, Wuhan, China; 2 School of Basic Medicine, Army Medical University (Third Military Medical University), Chongqing, China

**Keywords:** Post-translational modifications, Stress signalling, Diseases

## Abstract

HDAC3 is a specific and crucial member of the HDAC family. It is required for embryonic growth, development, and physiological function. The regulation of oxidative stress is an important factor in intracellular homeostasis and signal transduction. Currently, HDAC3 has been found to regulate several oxidative stress-related processes and molecules dependent on its deacetylase and non-enzymatic activities. In this review, we comprehensively summarize the knowledge of the relationship of HDAC3 with mitochondria function and metabolism, ROS-produced enzymes, antioxidant enzymes, and oxidative stress-associated transcription factors. We also discuss the role of HDAC3 and its inhibitors in some chronic cardiovascular, kidney, and neurodegenerative diseases. Due to the simultaneous existence of enzyme activity and non-enzyme activity, HDAC3 and the development of its selective inhibitors still need further exploration in the future.

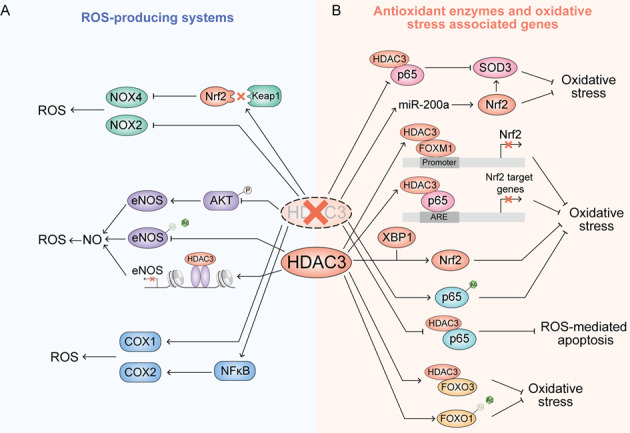

## Facts

- HDAC3 is critical for early embryonic growth, development of vital organs, and numerous metabolic processes.
- HDAC3 is closely correlated to mitochondria function and metabolism, ROS-produced enzymes, and antioxidant enzymes.
- Although current research on the function of HDAC3 is mainly focused on its deacetylase activity, its non-enzymatic functions are also considerable and worth investigating.

## Open questions

- What is the physiological function of HDAC3 in biological development and redox homeostasis?
- There seems to be some contradiction between the key role of HDAC3 in embryonic and organ development and its role as an accomplice in many chronic diseases. Is this contradiction related to its enzymatic and non-enzymatic activities?
- What are the central challenges of developing selective HDAC3 drugs targeting for oxidative stress?

## Introduction

Oxidative stress is a concept in redox biology and medicine, which is defined as an imbalance of homeostasis between the production and the removal of reactive oxygen/nitrogen species (ROS/RNS) via antioxidative protection systems [1, 2]. Cells always suffer the hazardous influence of endogenously or exogenously generated highly reactive oxidizing molecules. In normal conditions, these oxidants are formed in a controlled manner and act as important signaling molecules to regulate such processes as signal transduction, inflammation, immune function, autophagy, and stress responses [3]. However, excessive occurrence of oxidant will cause oxidative stress that damages cellular components such as proteins, lipids, and DNA and contributes to the pathogenesis and pathophysiology of numerous acute and chronic diseases and cancer [4]. The irreversible process of oxidative decay induced by ROS will also have a negative effect on the status of the biology of aging and reduce lifespan [1, 5].

**Fig. 1:**
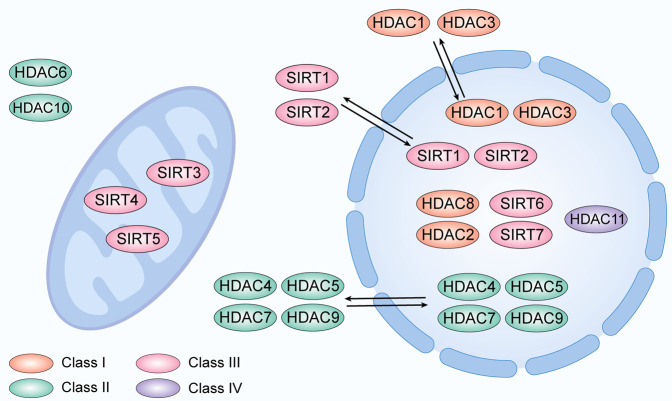
Cellular sub-localization of HDAC family proteins. Localization of Class I-IV HDAC proteins in cytoplasm, nucleus, and mitochondria.

Histone deacetylases (HDACs) are a group of enzymes, which reversibly regulate the acetylation level of histone and even nonhistone proteins in concert with histone acetyltransferases (HATs). In the mammalian genome, the HDAC superfamily consists of 11 HDAC isoforms. HDAC1, 2, 3, and 8 are classified as Class I HDACs which are mainly located in the nucleus. In particular, HDAC1 and HDAC3 can travel between the cytoplasm and nucleus [6, 7]. HDAC4, 5, 6, 7, 9, and 10 are classified as Class II HDACs. HDAC4, 5, 7, and 9, which contain only an N-terminal regulatory domain are classified as the Class IIa subfamily, which also can translocate between the cytoplasm and nucleus [8]. HDAC class IV currently only contains HDAC11, which possesses a conservated sequence within the deacetylase domain as well as with class I and II HDACs and is mainly expressed in the nucleus. Class III HDACs (also named sirtuins, including SIRT1, 2, 3, 4, 5, 6, and 7) have a unique deacetylase domain and are mainly expressed in the nucleus (SIRT1, 2, 6, 7) or mitochondria (SIRT3, 4, 5) [9, 10] (Fig. 1). Among them, HDAC3 is unique in that it forms a complex with silencing mediator of nuclear receptor co-repressor 1 (NCoR1) and retinoic acid and thyroid hormone receptor (SMRT; also known as NCoR2), which are nuclear receptor co-repressors and necessary to exert its catalytic activity [10–12]. HDAC3 also has important non-enzymatic functions, which are independent of NCOR1/2 nuclear receptor co-repressors [13]. Increasing evidences show that HDAC3 exhibits an important physiological function and is involved in regulating the redox system, including mitochondrial metabolism, modification of antioxidant genes, and ROS sensitivity [14–16].

## Overview of HDAC3

### The unique structure of HDAC3

All class I HDAC enzymes exhibit structural similarity to reduced potassium depenency 3 (RPD3) in yeast, and mainly contain a catalytic core domain and C- and N-terminals [17]. HDAC1 and HDAC2 were discovered in *Homo sapiens* firstly, then the homologous identification of HDAC3 indicated that HDAC3 also exhibits a resemblance to RPD3 proteins but possesses some characteristic structures compared to HDAC1 and HDAC2. The local position of some amino acid residues is unique in HDAC3. HDAC1 and HDAC2, which belong to the same class I HDACs as HDAC3, possess a glutamate at position 92, while the latter shows an aspartate residue at the same position. Phenylalanine is present at position 199 and replaces the original tyrosine residue in HDAC1 and HDAC2. The serine of HDAC1 and HDAC2 at position 107 is also replaced by tyrosine that generates a steric hindrance [18]. Besides these residue replacements, HDAC3 possesses some unique structural differences at positions 13 and 29 [19]. The presence of these residue replacements and dissimilarities generates the unique molecular structure and offers great potential for the creation of selective inhibitors of HDAC3.

### The essential functions of HDAC3

#### Enzymatic activity of HDAC3

**Fig. 2:**
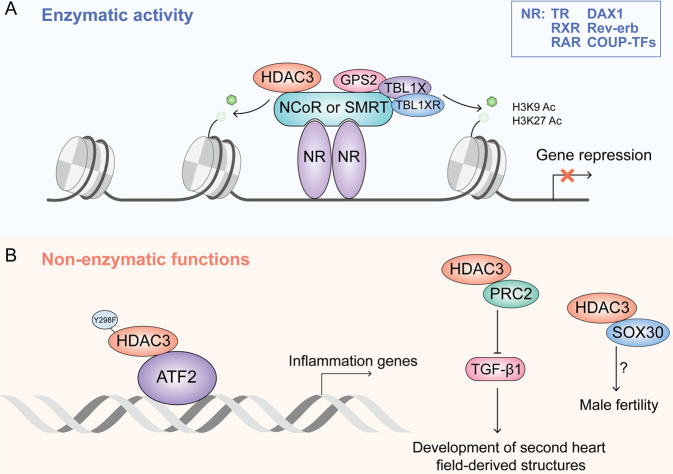
The enzymatic activity and non-enzymatic functions of HDAC3. **A** HDAC3 is recruited to interact with NCoR or SMRT, forming nuclear receptor co-repressors complexes with G protein pathway suppressor 2 (GPS2), transducin β-like 1 (TBL1X), and TBL1-related protein 1 (TBL1XR). Co-repressors complexes binds to NR that have bound chromatin to perform transcriptional regulation dependent on its deacetylation function. **B** HDAC3 mutated in deacetylation sites directly binds to ATF2 to regulate transcription of inflammation-related genes independently of deacetylase activity. HDAC3 also can interact with PRC2 or SOX30 to regulates heart development and male fertility.

As crucial switches of genetic transcription, nuclear receptors can integrate and deliver various signals in the organism, including developmental, nutrient, and stress signals, to the genome then regulate gene expression. Classically, a nuclear receptor will change structure after binding to the ligand and recruit co-repressors or co-activators, which can bind to ligand-free or ligand-bound nuclear receptors to regulate gene activation or repression, respectively [20]. However, in the absence of the ligand, nuclear receptors can also regulate gene expression via interacting on chromatin with NCoR or SMRT [21]. HDAC3 mainly has the enzymatic activity dependent on interacting with NCoR or SMRT, both of which can form a stable complex with HDAC3 to catalyze deacetylation and repress transcription. Current evidence indicates that HDAC3 has the ability to suppress specific nuclear receptors via cooperation with NCoR/SMRT, including retinoid X receptor (RXR), nuclear receptor subfamily 0 group B member 1 (NR0B1), thyroid hormone receptor (TR), nuclear receptor subfamily 1 group D member 2 (NR1D2, also known as Rev-erb), peroxisome proliferator-activated receptor (PPAR), retinoic acid receptor (RAR)and COUP-TFs [22] (Fig. 2A). Located at the N-terminus of SMRT and NcoR, the deacetylation activation domain (DAD) contains two SANT motifs (SANT1 and SANT2) that physically interact with activated HDAC3. SANT2, a highly conserved sequence motif for HDAC3 activation and binding, is a crucial component of histone interacting domain (HID) [23]. Intriguingly, HDAC3 in the unbound state is unstable and sequestered by T-complex 1 (TCP1), which is a ring complex in the cytoplasm [24, 25].

#### Non-enzymatic activity of HDAC3

HDAC3 also possess some functions, besides the classic enzyme activity. HDAC3 is vital for the growth of embryos and some organs, the global loss of which is lethal [10, 25]. Some studies have indicated that the deacetylase activity of HDAC3 can be removed via mutating some core site (Y298F). Unexpectedly, these proteins without enzyme activity still partially rescue the effect of Hdac3 absence. Recent evidence shows that HDAC3 selectively binds to ATF3 in a manner dependent on its deacetylase activity to inhibit Toll-like receptor signaling, but can also activate transcription factor 2 (ATF2) independent of NCoR1/2 and activate the inflammatory genes. HDAC3 acts as a dichotomous transcriptional activator or repressor to regulate the innate immune system via non-enzymatic functions [13]. It can recruit polycomb repressive complex 2 (PRC2) which is activated to regulate the transfer of histone methyl and promote epigenetic silencing of transforming growth factor-β1 (TGF-β1) which is responsible to affect the development of second heart field-derived structures [26]. HDAC3 can also coordinate with SRY-box transcription factor 30 (SOX30) to control male fertility independent of its enzymatic activity during meiotic exit of spermatogenesis, but the detailed mechanism is unclear [27] (Fig. 2B).

#### Physiological functions of HDAC3

Given that whole-body deletion of HDAC3 leads to early embryonic lethality, researchers have constructed a number of tissue-specific HDAC3 knockout mice via the Cre recombinase (Cre) system, gradually unraveling the physiological functions of HDAC3. In heart, the absence of HDAC3 in the embryo results in severe hypertrophic cardiomyopathy and lethality by 3 to 4 months [28]. In the developing murine epicardium, the loss of HDAC3 causes ventricular myocardial wall hypoplasia with reduction of epicardium-derived cells [29]. In neural progenitor cells (NPCs) of the central nervous system, HDAC3 deficiency can impair neuronal migration and cortical lamination, resulting in serious lethality within 16 hours after birth [30]. Loss of HDAC3 in lung endodermal epithelium impairs early alveologenesis, resulting in disruption of lung sacculation and lethality between 2 and 10 days [31]. HDAC3-deficient alveolar macrophages (AMs) also exhibit serious mitochondrial oxidative dysfunction and homeostasis disorders [14]. In the intestine, HDAC3 can mobilize intestinal lymphocytes to defend against pathogenic microorganisms and maintain intestinal homeostasis [32]. In the reproductive system, loss of HDAC3 can cause nonreceptive endometrium and female infertility [33]. Moreover, HDAC3 contributes to the development and physiological remodeling of adipose tissue and bones, skeletal muscle metabolism, glucose-stimulated insulin secretion and immune responses of several immune cells [10]. In white adipose tissue (WAT), the selective ablation of HDAC3 can switch the metabolic signature of WAT, potentiates WAT oxidative capacity, and promote browning [34]. HDAC3 is also required for maintaining body temperature in response to acute cold exposure in brown adipose tissue (BAT), the lack of HDAC3 resulting in downregulation of mitochondrial oxidative phosphorylation and diminution of mitochondrial respiration [35]. Similar to the above, the role of HDAC3 in regulating skeletal muscle metabolism and contractile function still cannot be ignored. HDAC3-depleted muscles display decreased glucose utilization, increased branched-chain amino acid (BCAA) catabolism, and severe systemic insulin resistance, resulting in reduced muscle mass during ageing [36]. According to known studies, liver-specific deletion of HDAC3 leads to steatosis and accumulation [37], accumulated DNA damage, chronic liver injury, metabolic disorder, and transcriptional reprogramming [38]. This revealed the indispensable role of HDAC3 in the regulation of hepatic lipid metabolism. Given the intricate tissue-specific function of HDAC3, it plays vital roles in numerous organ injuries and diseases, which are summarized in our previous review [39].

## HDAC3 regulate oxidative stress

Oxidative stress is characterized as the imbalance between the production and scavenging of reactive oxidants. Reactive oxidants are generated from various sources in several compartments under physiological or pathologic conditions. Recent extensive studies suggested that HDAC3 can regulate oxidative stress via multiple pathways. The regulation of oxidative stress by HDAC3 is also controversial due to differences in the targets and ways.

### HDAC3 regulates mitochondrial metabolism and function

Mitochondria have a dominant role in the generation of free radicals. As a primary place of ROS production under physiological and pathophysiological conditions, mitochondria continuously utilize molecular oxygen as a substrate to produce ROS either intentionally or as accessory substances under the action of many enzymes, including microsomal cytochrome P450 (CYP), plasma membrane-bound NADPH oxidase and cytoplasmic xanthine oxidase. During the normal process of mitochondrial respiration, a fraction of the electrons, which originates from the electron transport chain within ATP synthesis, escape to oxygen impulsively and generate the oxygen free radical superoxide. It is estimated that 2% of all electrons in the electron transport chain is reduced to form superoxide radicals instead of transforming oxygen into water [40].

The regulation of mitochondrial function and metabolism by HDAC3 is controversial, depending on the target genes interaction with HDAC3 and tissue specificity. In skeletal muscle tissue and the heart, the loss of HDAC3 causes abnormalities of mitochondrial metabolic processes, including tricarboxylic acid cycle (TCA), ATP synthesis, the electron transport chain (ETC) and even fatty acid metaboli, resulting in mice being more sensitive to a high fat diet and more susceptible to cardiomyopathy [41]. It is suggested that HDAC3 is crucial in the upkeep of mitochondrial function. The mitochondria from BAT with HDAC3 loss also exhibit impaired substrate-induced respiration, with impaired function of complexes I, II, and IV of the ETC [35]. In consistently, HDAC3 can control mitochondria function in lung; the genes involved in oxidative phosphorylation and subunits of complexes I–V of the ETC are significantly downregulated in HDAC3-deficient AMs. In HDAC3-cKO AMs, the number of mitochondria and mitochondrial membrane potential (MMP) significantly reduced. Further research has shown that HDAC3 deficiency induces mitochondrial dysfunction associated with impaired PPARγ signaling [14]. Activated PPARγ forms a complex through heterodimerization with RXR, then translocates to the nucleus for binding with promotor of PPAR response elements (PPREs), resulting in the regulation of the transcription of target genes [42]. Meanwhile, PPARγ, together with its coactivator PGC-1α, is a robust regulated factor for mitochondrial biogenesis via activating several nuclear transcription factors (such as TFAM, NRF1, NRF2, YY1, and SP-1), which are important in activating the formation and assembly of mitochondria [43]. Inhibition of PGC-1α and PPARγ leads to a reduced capacity for mitochondrial oxidative phosphorylation (OXPHOS) and increased ROS production [44]. The phosphorylation of HDAC3 can increase the binding between PINK1 and p53, resulting in hypoacetylation of p53, enhancing mitophagy, and suppressing apoptosis in the inflammation cell [45]. However, HDAC3 and NCoR complex has also been found to reduce intracellular heme resulting from the transcription repression of PGC-1α, whereby mitochondrial respiration is disrupted in a heme-dependent manner [46]. It is not clear whether this contradiction is due to the tissue-specific function of HDAC3 or other mechanisms.

Metabolic cues play a potential role in configuring mitochondrial dynamics and function, accumulation of free fatty acids in mitochondria can induce an increase of mitochondrial ROS (mtROS) and impair mitophagy; fatty acid β-oxidation (FAO) is critical for scavenging superfluous mtROS and reducing mitochondrial damage under cellular stress [47]. In LPS-stimulated macrophages, HDAC3 can translocate to the mitochondria to deacetylate the lysine 303 of hydroxyacyl-CoA dehydrogenase trifunctional multienzyme complex subunit α (HADHA), which is a crucial rate-limiting enzyme for FAO, to repress its enzyme activity and restricts FAO-mediated OXPHOS [48].

**Fig. 3:**
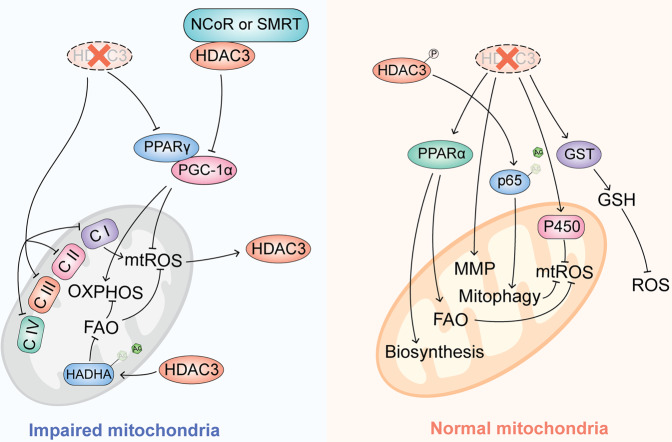
HDAC3 regulate mitochondrial metabolism and function. The role of HDAC3 in mitochondria and regulation of mtROS is complex and fraught with contradictions. HDAC3 can both impair and protect mitochondria through regulating different signaling pathways, mtROS, FAO, OXPHOS, and mitophagy.

In contrast, in intestinal epithelial cells and liver cells, deletion or pharmacological inhibition of HDAC3 can promote mitochondrial biosynthesis and FAO via regulation PPARα [49, 50]. The deletion of HDAC3 in intestinal epithelial cells can significantly upregulate members of the glutathione-S-transferase (GST) and cytochrome P450 gene families [49], which play a potential role in scavenging ROS and detoxifying xenobiotics [51]. In a mouse kidney transplantation model and hepatocellular carcinoma (HCC), the inhibition of HDAC3 can maintain MMP and reduce mitochondria-related apoptosis [52, 53]. Interestingly, mtROS also appears to influence HDAC3 activity reversely: the mtROS from complex I can activate HDAC3 and promote deacetylation of p65, resulting in a reduction of TNF-α in cardiomyocytes [54]. However, the exact mechanism of this regulation and whether it is tissue-specific remains unknown. HDAC3 seems to play a role of a double-edged sword in mitochondria function through regulating different target genes or pathways (Fig. 3). Although the relationship between HDAC3 and mitochondria still wrap in the mist, HDAC3 indeed can regulate mitochondrial metabolism and function through its enzymatic activity or unknown non-enzymatic functions, acting as an indispensable regulator of cellular ROS generation and oxidative stress status.

### HDAC3 regulates the endoplasmic reticulum

In addition to mitochondria and peroxisomes, ROS also originate from the endoplasmic reticulum (ER). In ER, cytochrome P450 family enzymes and the interaction of endoplasmic reticulum oxidoreductin-1 (ERO1) with protein disulfide isomerase (PDI) are the main contributors of ROS production [55]. During ERO1 oxidation of proteins, electrons are transferred from PDI to molecular oxygen and proteins undergo several thiol-disulfide exchange reactions during disulfide bond modifications, triggering the generation of ROS and contributing to ER stress [56]. An estimation analysis revealed that about 25% of ROS are produced during the process of oxidative protein folding [57]. A recent study showed that HDAC3 can transactivate the KDEL (Lys‐Asp‐Glu‐Leu) receptor (KDELR2), a crucial ER stress‐responsive and ER homeostasis maintenance factor, accelerating cell cycle progression and the proliferation of breast cancer by protecting the ER traffic transport protein POC5 from degradation [58]. Selective inhibition of HDAC3 can alleviate β-cell dysfunction and apoptosis by repressing proapoptotic ER stress [59, 60]. Similarly, hypoxia-reoxygenation increases HDAC3 expression and ER stress in cardiomyoblasts, but the inhibition of HDAC3 can significantly attenuate ER stress-associated apoptosis [61]. In summary, there are relatively limited studies on HDAC3 and ER and ER-associated ROS, and whether HDAC3 can directly regulate ER function and protein folding needs to be further explored in the future.

### HDAC3 regulates ROS-producing systems

#### NADPH oxidases

Nicotinamide adenine dinucleotide phosphate (NAPDH) oxidases (NOXs) are a family of membrane-bound enzyme complexes that catalyze the transfer of electrons across biological membranes. NOX is one of the major sources of cellular ROS [62]. In recent years, seven NOXs isoform (NOX1-NOX5, dual oxidase 1 (DUOX1), and DUOX2) have been identified [63]. Silencing of HDAC3 impairs the interaction of KEAP1 and Nrf2, increasing the Nrf2 level, resulting in a decrease of NOX4 [15]. Consistently, the knockdown of HDAC3 in vitro represses the combination of transcription factors and polymerases with the NOX4 promoter, thereby inhibiting transcription of NOX4 and production of NOX4 associated ROS in human endothelial cells [64]. In mice with spinal cord injury, inhibition of HDAC3 can reduce NOX2 and NOX4 protein expression and attenuate ROS production [65]. The relationship between HDAC3 and NOXs need further exploration.

#### Nitric oxide synthases

Nitric oxide synthases (NOSs) are a family of enzymes catalyzing L-arginine into nitric oxide (NO), which acts as a key gaseous signaling molecule and biological messenger like ROS at low levels, participating in many important biological processes [66]. However, abnormal production of NO can cause oxidative stress as well as nitrative stress by producing peroxynitrite and nitrosating species, which can combine with oxygen and trigger impairments of proteins, lipids, and DNA [67]. In mammals there mainly exist three NOS isozymes, namely neuronal (nNOS or NOS I), inducible (iNOS or NOS II), and endothelial (eNOS or NOS III) NOS, which are distributed in different tissue cells and perform a variety of functions. eNOS is mainly expressed in endothelial cells and regulated by agonists such as acetylcholine and bradykinin and stress of the flowing blood [68]. In cardiovascular tissue, the lysine acetylation of eNOS can stimulate its enzymatic activity and increase the production of NO. The appropriate generation of NO can improve endothelium-dependent vascular function [69]. A previous study indicated that HDAC3 is an important factor in endothelial integrity and regulates AKT phosphorylation and activity [70]. The inhibition of HDAC3 will suppress AKT-eNOS signaling and generation of NO. HDAC3 has also been found to deacetylate the lysine of eNOS, inhibiting its activity and antagonizing its improvement of vascular function [71]. Besides deacetylated lysine, HDAC3 can also be recruited to the core promoter region of eNOS and induce histone deacetylation, which represses eNOS transcriptional activity [72]. HDAC3 also inhibits iNOS expression and NO generation via PI3K/Akt pathways or PPARγ [16, 73].

#### Cyclooxygenases

**Fig. 4:**
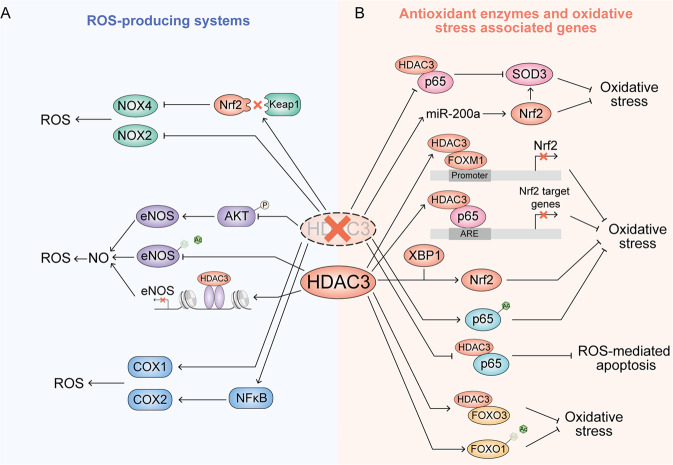
HDAC3 regulation oxidative stress-associated enzyme and genes. **A** HDAC3 regulates the enzymes responsible for ROS production. **B** HDAC3 regulates antioxidant enzymes and oxidative stress-associated transcription factor.

Cyclooxygenases (COXs), also known as prostaglandin-endoperoxide synthase (PTGSs), are enzymes responsible for catalyzing the conversion from arachidonic acid to prostanoids, producing ROS as a byproduct during this process [74, 75]. Although there is some evidence that COX can modulate ROS generation, the relationship of HDAC3 and COX is mainly focused on the regulation of its immune and inflammation-related functions. In HDAC3-deficient macrophages, the promoter and an upstream enhancer region of COX-1 are significantly hyperacetylated and COX-1 is strongly overexpressed [76]. The inhibition of HDAC3 also can induce COX-2 expression via the NF-κB pathway [77] or binding with COX-2 promoter sequences [78] (Fig. 4A).

### HDAC3 regulates antioxidant enzymes and oxidative stress-associated genes

#### Superoxide dismutases

Superoxide dismutases (SODs) are universal enzymes that alternately catalyze the dismutation of superoxide into ordinary oxygen and hydrogen peroxide. SODs are important to maintain the levels of a variety of ROS and RNS, and limit their potential toxicity and control broad aspects of cellular redox biology via signaling functions [79]. In mammalian tissues, there are three SODs isoforms: SOD1 (copper/zinc-SOD), SOD2, and SOD3 (extracellular SOD) [80]. As a key vascular antioxidant enzyme, SOD3 plays a dominant role in protecting of the pulmonary circulation against oxidative stress. The intracellular level of SOD3 is tightly regulated by HATs and HDACs. HDAC3 inhibition can significantly promote SOD3 expression which is beneficial to the alleviation of idiopathic pulmonary arterial hypertension (IPAH) [81]. The inhibitor of HDAC3 can also increase the SOD level through inhibiting nuclear translocation of HDAC3 [82, 83]. However, the mechanism of HDAC3 regulation of SODs is still unclear; whether the inhibition of HDAC3 induced increase of SOD is directly a function of HDAC3 activity or an outcome of inhibiting inflammation is unknown.

#### Nrf2

Nuclear factor erythroid 2-related factor 2 (Nrf2), one of the master regulators of cellular antioxidant response, is expressed in almost the whole body and maintained at a low baseline level in the absence of stress [84]. Nrf2 is a leucine zipper (bZIP) protein which consists of seven Neh domains. The antioxidative function of Nrf2 is mainly derived from its capacity to transcriptionally regulate more than 200 target genes possessing antioxidant response elements (AREs), which are involved in redox homeostasis, energetic metabolism, iron and heme metabolism, proliferation, cell death, proteasomal degradation and mitochondrial physiology [85]. The protein level of Nrf2 is negatively regulated by three E3 ubiquitin ligase complexes: HRD1, KEAP1-CUL3-RBX1 complex, and the β-TrCP-SKP1-CUL1-RBX1 complex. They degrade Nrf2 protein in a ubiquitin-dependent manner by binding with different Neh domains. The regulation of Nrf2 by HDAC3 occurs mainly indirectly, in a manner dependent on its HDAC. In pulmonary fibrosis (PF) mice, HDAC3 combines with the profibrotic transcriptional factor forkhead box protein M1 (FOXM1) and binds to Nrf2 promoter, resulting in a decrease of local histone 3 acetylation and repression of Nrf2 and its transcription. Insufficient Nrf2 and enzymatic antioxidants, such as catalase and SOD3, impairs antioxidant stress and antifibrosis capacity in lung tissue [86]. Besides FOXM1, p65 has also been discovered to cooperate with HDAC3 to transcriptionally repress the Nrf2-ARE pathway and promote oxidative stress-induced necrosis [87, 88]. Inhibition of HDAC3 via siRNA or a specific inhibitor can activate Nrf2 through decreasing Keap1-Nrf2 interaction, promoting downstream NOX4 redox signaling in the vascular endothelium. Finally, the inhibition of HDAC3 reduces ROS production, endothelial impairment and alleviates endothelial injury caused by type 2 diabetes mellitus (T2DM) [15]. Similarly, the inhibition of HDAC3 can also increase miR-200a and activation of Nrf2 via inhibiting the interaction between Keap1 and Nrf2 protein [89]. Interestingly, some studies have found the opposite outcome and suggest that X-box-binding Protein 1 (XBP1) increases nuclear localization of Nrf2 and activates HO-1 via HDAC3-dependent ways; the overexpression of XBP1 can increase Nrf2 and protect endothelial cells from oxidative stress [90]. HDAC3 has also been found to protect endothelial cells against apoptosis due to atherosclerosis [70].

#### NF-κB

NF-κB is a family of transcription factors regulating the transcription of several genes that are involved in cell growth, development, differentiation, and death. NF-κB is crucial in the regulation of oxidant stress, inflammation, and immunity [91]. NF-κB protein is a complex containing five different related family members all having a Rel-homology (RHD) domain to bind to DNA or other proteins [92]. The members of NF-κB can be divided into two classes. In Class I, both p50 and p52 are processed from the larger precursor proteins p105 and p100 by proteasome and contain a C-terminal ankyrin repeats. In Class II, the other three members, RelA (also known as p65), RelB, and cRel can positively regulate gene transcription with a C-terminal transcription activation domain (TAD) [93]. As mentioned previously, excess accumulation of ROS will cause serious oxidative damage and even cell death. Abundant evidence indicates that NF-κB can attenuate or promote ROS through regulation of different target genes [93]. NF-κB has been reported to be activated via various post-translational modifications. The most famous subunit, p65, can be modified by phosphorylation, acetylation, ubiquitination, and monomethylation [94–97]. HDAC3 can directly modify lysine at different sites of p65 to control its transcriptional activity in vitro and vivo tightly and distinctly. A previous study indicated that the acetylation of p65 occurs at three major sites: lysines 218, 221, and 310 [91]. In bacterially infected macrophages, homeodomain-interacting protein kinase 2 (HIPK2) interacts with HDAC3, promoting HDAC3 phosphorylation at S374 and inhibiting HDAC3 deacetylase activity, resulting in p65 acetylation at K218 to alleviate the tissue injury from immoderate oxidative stress and inflammation [98]. The inhibitors of HDAC3 cause macrophages to exhibit anti-inflammatory properties via inhibiting p65 transcriptional activity [99, 100]. In osteoclasts, HDAC3 is reported to deacetylate p65 and deletion of HDAC3 increases p65 acetylation at K310 and transcriptional activity, resulting in impairment of bone modeling [101]. In intestinal tissue, retinoic acid-related orphan receptor α (RORα) and HDAC3 compose the transcriptional corepressor complex that significantly suppresses transcriptional activity of NF-κB and attenuates intestinal inflammation [102]. A recent study also found that HDAC3 also deacetylates p65 at K122 and K123, promoting transcription of cGAS [103]. Specifically, mimical acetylation mutations of p65 at lysine 221 induces a sharp decline in the overall DNA binding activity of p65 homodimers [96]. However, whether HDAC3 can deacetylate p65 at K221 remains unclear. In addition, HDAC3 has also been found to interact with p65 and translocate into the nucleus together. The inhibition of interaction and nuclear translocation of p65 with HDAC3 can repress excessive differentiation and proliferation of keratinocytes, which depends on reducing ROS-mediated apoptosis with loss of MMP [104]. Similarly, the inhibition of the nuclear translocation of HDAC3 and p65 in bronchial epithelial cells can also reduce oxidative stress and mtROS to protect against endotoxin-induced acute lung jury [6].

#### FOXOs

Forkhead box O proteins (FOXOs) are a subclass of the forkhead transcription factors [105], mainly including four members FOXO1, FOXO3, FOXO4, and FOXO6. FOXOs play a crucial role in cellular stress response and antioxidant defencse. Specifically, FOXOs are observed to contribute to determining lifespan, increased FOXO activity resulting in extended lifespan in model organisms [106]. ROS can also affect FOXO activity in multiple ways, including posttranslational phosphorylation and acetylation modifications, adjustment of subcellular localization, interaction with some coregulators, alternation of protein synthesis and stability [107]. FOXOs indorse the transcription of genes coding for antioxidant proteins located in different intracellular compartments, including SOD2, HO-1, peroxiredoxin-3 (Prx3), GPX1, and thioredoxin reductase (TrxR2) [107]. Usually, deacetylation of FOXOs can increase their transcriptional activity and affect the expression of genes that are dependent, which is responsible for additional ROS detoxification [108]. Multiple lysine acetylation sites of FOXOs have been identified in FOXO1 (K245, K248, K262, K274, K294, K559), FOXO3 (K242, K245, K259, K271, K290, K569), FoxO4 (K186, K189, K215, K237, K407), and FOXO6 (K173, K176, K190, K202, K229) [109]. In neurons, HDAC3 can co-locate with FOXO3 at promoters of target genes and increase binding affinity to DNA, and the absence of HDAC3 can cause abnormal locomotor behavior [110]. FOXO1 and FOXO3 have also been identified as direct deacetylation targets of HDAC3 in the liver [111]. The deacetylation of FOXO1 can protect against oxidative stress-induced acute β-cell failure to preserve insulin biosynthesis and secretion (Fig. 4). In addition, there is a definite and detectable relationship existed between sirtuins and deacetylation of FOXOs. FOXO1 and FOXO4 are deacetylated by SIRT1, causing their nuclear translocation and increasing transcription of the antioxidant enzyme SOD-2 against oxidative stress [112, 113] (Fig. 4B).

## The role of HDAC3 and its inhibitors in chronic diseases

### Cardiovascular diseases

In the pathophysiology of the heart, oxidative stress plays a pivotal role. Several studies have revealed the overproduction of ROS and RNS as one of the most common pathological features of ischemic myocardial damage and closely associated with subsequent cardiac dysfunction [114, 115]. A major cause of ischemic cardiomyopathy, myocardial infarction (MI), is mainly caused by coronary artery obstruction and immediately hampers the oxygen and nutrient supplies of the myocardium, causing cardiomyocyte death and heart tissue damage [116]. In diabetic rats MI models, HDAC3 is significantly upregulated and cardiocyte-specific HDAC3 deletion can attenuate diabetic MI through the Rev-erbα/BMAL1 pathway to activate mitophagy [39]. HDAC3 can also interact with p65 to repress Nrf2-ARE transcriptional activity and the expression of antioxidant proteins, aggravating the tissue damage of MI [87]. Besides that, recent studies have revealed that hypoxia can increase the level of the long noncoding RNA taurine upregulated gene 1 (TUG1), which can reduce miR-132-3p and increase HDAC3, which decreases the acetylation of H3K9 and epigenetically represses the transcription of antioxidative genes, endorsing the production of ROS and the pathogenic deteriorate of MI [117]. Cyclin-dependent kinase 2 (CDK2) is a serine/threonine kinase, of which the abnormal activation is related to MI [118]. RGFP966, a selective inhibitor of HDAC3, can reduce the CDK2 of the myocardium through increasing miR-19a-3p expression, alleviating oxidative stress and even cardiac injury after MI [119]. Besides cardiomyocytes, the recruitment and phenotypic differentiation of macrophages are also important for myocardial repair and functional remodeling during MI. A recent study indicated that transcriptional coactivator with PDZ-binding motif (TAZ) and Yes-associated protein (YAP) can interact with HDAC3-NCoR1 to inform a repressor complex to repress arginase-I (Arg1) expression and increase interleukin 6 (IL6) expression leading to the impedance of reparative response. The deletion of YAP/TAZ or pharmacological inhibition of HDAC3 can decrease hypertrophy and fibrosis, and improve angiogenesis which brings out the improved cardiac function after MI [120]. What’s more, the structural and functional changes caused by adverse cardiac remodeling after MI leads to heart failure (HF). MPT0E014, an inhibitor of HDAC, has been proved to alleviate cardiac hypertrophy, cardiac fibrosis and structural remodeling and improve cardiac contractility [121]. HDAC3 can deacetylate DNMT1 to repress SHP-1 expression, promoting the development of cardiomyocyte hypertrophy-induced HF [122]. A decrease of HDAC3 is also associated with restoring cardiac function with left ventricular HF [123]. Silencing of HDAC3 confers protection against HF by inhibiting miR-18a-targeted ADRB3 in cardiomyocytes [124]. Endothelial dysfunction is another key factor for diabetic microvascular disease, diabetic cardiomyopathy, and HF. In high glucose-induced endothelial cells, β-hydroxybutyrate suspends the colocalization of HDAC3 with β-catenin through acetylating H3K14 in the promoter of claudin-5, alleviating cardiac microvascular hyperpermeability [125]. Interestingly, although most of the current studies show that HDAC3 seems to play an evil role in many cardiovascular diseases, and pharmacological or genetic inhibition of HDAC3 can improve MI, myocardial fibrosis, and myocardial remodeling, HDAC3 also is essential for heart development. Many studies have proved that the deletion of HDAC3 causes the loss of ventricular structure and developmental abnormalities of the heart [29, 126]. This seemingly contradictory conclusion is associated with the important non-enzymatic action of HDAC3, some studies also finding that HDAC3 deacetylase activity is dispensable for repression of myocyte differentiation [126]. However, its non-enzymatic activity is essential for the differentiation of cardiac stem cells although the exact mechanism is still unclear.

### Kidney diseases

Renal fibrosis is an inevitable pathohistological characteristic when renal ageing and chronic kidney disease (CKD) occurs [127]. Similar to fibrosis in other organs, renal fibrosis is essentially an overhealing/repairing process. Particularly, normal kidney cells undergo various kinds of damage, then gradually lose their original phenotype, and transdifferentiate to myofibroblasts, which leads to excessive production and deposition of extracellular matrix (ECM), which finally causes irreversible structural damage and dysfunction of the kidney [128]. Klotho is an anti-ageing protein mainly expressed in the kidney. A decrease of Klotho is closely correlated with the progression of CKD [129]. In various fibrotic kidney models, HDAC3 is upregulated with concomitant suppression of Klotho, whereas deletion of HDAC3 significantly increases resistance to fibrosis. Further research has shown that HDAC3 can interact with the transcription activator NCoR and NF-κB and bound to the promotor of Klotho [130]. TGF-β is generally regarded as an important regulator of multiple chronic fibrotic diseases via either Smad-dependent or Smad-independent signaling pathways [131, 132]. In kidney podocytes, TGF-β can recruit HDAC3 and NCoR complex to down-regulates miR-30 then induce podocyte injury, and RGFP966 can alleviate the podocyte cytoskeleton damage [133].

Current multiple studies have reported that histone acetylation is a common and critical epigenetic modification in kidney development and pathogenesis. The suppression of HDACs can exert anti-inflammation and anti-fibrotic functions in numerous kidney disease models. It has been indicated recently that HDAC2 is upregulated by TGF-β in diabetic kidney [134] and HDAC4 selectively contributes to podocyte injury in diabetic nephropathy [135]. The pan-HDAC inhibitor trichostatin has also been proved to block TGF-β1-induced epithelial-mesenchymal transition (EMT) in renal epithelial cells contributing to the treat of IRI-induced renal fibrosis [136].

### Neurodegenerative diseases

Neurodegenerative diseases are a set of hereditary or sporadic conditions characterized by progressive degeneration of neurons in specific regions of the brain [137]. Accumulating evidence points to HDACs and epigenetic mechanisms being important regulators of brain development and degeneration process. HDACs was first found to play an important role in neurodegenerative disease in a study in 2001, which indicated that pharmacological inhibition of HDACs can suppress polyglutamine-dependent neurotoxicity in *Drosophila* [138]. A growing and compelling body of evidence points to HDAC3 being a particularly critical regulator in the promotion of neurodegeneration in various disease models. The knockdown of Had-3, the orthologue of mammalian HDAC3, can inhibit neurotoxicity resulting from the expression of a human huntingtin (Htt) fragment [139]. Recently, HDAC3 inhibitors have been proved to suppress degeneration and improve behavioral performance in multiple neurodegenerative conditions, including Parkinson’s disease (PD), Alzheimer’s disease (AD), Friedreich’s ataxia, spinal cord injury, and ischemic stroke [140–144]. AD is a common progressive neurodegenerative disease characterized by age-related dementia among the elderly. The deposition of amyloid-β (Aβ) protein is considered to be one of the most important neuropathological hallmarks, although the exact mechanism of AD is still unclear [145]. Recent studies have reported that 6‐ and 9‐month‐old APP/PS1 mice showed the higher level of HDAC3 in the hippocampus compared with age‐matched wild‐type mice, and the level of Aβ is increased in the hippocampus when HDAC3 is overexpressed. Furthermore, inhibition of HDAC3 can attenuate the deficit of spatial memory in APP/PS1 mice [146]. Consistently, inhibition of HDAC3 has also been proved to reverse pathological tau phosphorylation at Thr181, Ser202, and Ser396, promote Aβ degradation and decrease the accumulation of Aβ1–42 protein levels in the brain and periphery, and significantly improves spatial memory in another triple transgenic AD mouse model (3xTg-AD) [140]. PD is the most common central nervous system (CNS) disorder and is mainly related with progressive memory impairment and motor performance. Previous studies have demonstrated that histone deacetylase inhibitors can effectively relieve PD, but the precise mechanism is still unclear [147].

## Selective HDAC3 inhibitors

More and more inhibitors targeting HDACs have been discovered or synthesized recently, some of which (such as vorinostat (SAHA), belinostat, panobinostat, and romidepsin) have been approved by the U.S. Food and Drug Administration (FDA) for oncotherapy [148–151]. Unfortunately, most of these inhibitors do not target the activity of HDAC1, HDAC2, and HDAC3. Considering the critical character of HDAC3 in several pathophysiology conditions, selective pharmacological inhibition of HDAC3 becomes a potential therapeutic strategy. There are several types of HDAC3 inhibitors produced based on different compounds, such as benzamides, hydroxamates, hydrazides, and thiols [17]. Similar to other class I HDACs, HDAC3 also has a zinc-binding group, which is typically the binding site with small molecule inhibitors. Besides that, many HDAC inhibitors can mimic the lysine alkyl side chain dependent on a linker connecting the zinc-binding group to a capping group. The most common zinc-binding group contains o-aminoanilide and hydroxamic acid [39], and many selective HDAC3 inhibitor are designed based on their modification, such as RGFP966 and Entinostat (MS-275). PD106 and RGFP109 are two drugs developed to target Friedreich’s ataxia, which also have good selective potency for HDAC3 [152, 153]. BRD3308 was a new selective HDAC3 inhibitors and designed by referring to the clinically experienced HDAC inhibitor CI-994. In diabetes models, BRD3308 can inhibit the synthesis and secretion of inflammatory factors and reduce pancreatic β-cell apoptosis via selective inhibition of HDAC3 [154].In addition, several selective HDAC3 inhibitors derived from natural compounds have also been found [155, 156]. However, isoenzyme-selective HDAC inhibitors are infrequent: all the HDAC3 inhibitors mentioned above are o-aminoanilide derivatives, and the selectivity of most of these compounds is evaluated by measuring the IC_50_ [19]. More content about the development strategy of selective HDAC3 inhibitors that brings a huge challenge has been detailed in other molecular synthesis-related reviews [19, 157].

## Future perspective and conclusion

As an important and unique member of the HDAC family, HDAC3 plays a critical role in embryo and organ development, physiology, metabolism, and oxidative stress dependent on its enzymatic and non-enzymatic functions. In this review, we systematically summarize the important function of HDAC3 aiming at oxidative stress through regulation of mitochondria function and metabolism, ROS-produced enzymes, antioxidant enzymes, and oxidative stress-associated transcription factors. Although the relationship between HDAC3 and mitochondrial function and metabolism still wrap in the mist because of the complexity of HDAC3 functions and limited studies, it will be a direction worth exploring in the future.

Another burning question is the development of more specific HDAC3 inhibitors via distinguishing the high structural similarity of zinc-dependent HDAC isoenzymes. Although selective HDAC3 inhibitors, represented by RGF966, have been demonstrated to seemingly be a potential protective agent in some chronic diseases such as cardiovascular diseases, kidney disease, and neurodegenerative diseases, the precise mechanism still needs to be explored in depth. Moreover, due to the simultaneous existence of enzyme activity and non-enzyme activity, attention should be paid to distinguishing the regulatory diversity of these two functions in the development of drugs targeting HDAC3.

## Data Availability

The authors confirm that the data supporting the findings of this study are available within the article.

## References

[1] Pisoschi AM, Pop A (2015). The role of antioxidants in the chemistry of oxidative stress: a review. Eur J Med. Chem.

[2] Sies H (2015). Oxidative stress: a concept in redox biology and medicine. Redox Biol.

[3] Ma Q (2013). Role of Nrf2 in oxidative stress and toxicity. Annu Rev Pharm Toxicol.

[4] Gorrini C, Harris IS, Mak TW (2013). Modulation of oxidative stress as an anticancer strategy. Nat Rev Drug Disco.

[5] Maulik N, McFadden D, Otani H, Thirunavukkarasu M, Parinandi NL (2013). Antioxidants in longevity and medicine. Oxid Med Cell Longev.

[6] Pooladanda V, Thatikonda S, Bale S, Pattnaik B, Sigalapalli DK, Bathini NB (2019). Nimbolide protects against endotoxin-induced acute respiratory distress syndrome by inhibiting TNF-α mediated NF-κB and HDAC-3 nuclear translocation. Cell Death Dis.

[7] Kim JY, Shen S, Dietz K, He Y, Howell O, Reynolds R (2010). HDAC1 nuclear export induced by pathological conditions is essential for the onset of axonal damage. Nat Neurosci.

[8] Gray SG, Ekström TJ (2001). The human histone deacetylase family. Exp Cell Res.

[9] Zhang L, Han Y, Jiang Q, Wang C, Chen X, Li X (2015). Trend of histone deacetylase inhibitors in cancer therapy: isoform selectivity or multitargeted strategy. Med Res Rev.

[10] Emmett MJ, Lazar MA (2019). Integrative regulation of physiology by histone deacetylase 3. Nat Rev Mol Cell Biol.

[11] You S-H, Lim H-W, Sun Z, Broache M, Won K-J, Lazar MA (2013). Nuclear receptor co-repressors are required for the histone-deacetylase activity of HDAC3 in vivo. Nat Struct Mol Biol.

[12] Guenther MG, Barak O, Lazar MA (2001). The SMRT and N-CoR corepressors are activating cofactors for histone deacetylase 3. Mol Cell Biol.

[13] Nguyen HCB, Adlanmerini M, Hauck AK, Lazar MA (2020). Dichotomous engagement of HDAC3 activity governs inflammatory responses. Nature.

[14] Yao Y, Liu Q, Adrianto I, Wu X, Glassbrook J, Khalasawi N (2020). Histone deacetylase 3 controls lung alveolar macrophage development and homeostasis. Nat Commun.

[15] Huang S, Chen G, Sun J, Chen Y, Wang N, Dong Y (2021). Histone deacetylase 3 inhibition alleviates type 2 diabetes mellitus-induced endothelial dysfunction via Nrf2. Cell Commun Signal.

[16] de la Vega L, Grishina I, Moreno R, Krüger M, Braun T, Schmitz ML (2012). A redox-regulated SUMO/acetylation switch of HIPK2 controls the survival threshold to oxidative stress. Mol Cell.

[17] Adhikari N, Jha T, Ghosh B (2021). Dissecting histone deacetylase 3 in multiple disease conditions: selective inhibition as a promising therapeutic strategy. J Med Chem.

[18] Sarkar R, Banerjee S, Amin SA, Adhikari N, Jha T (2020). Histone deacetylase 3 (HDAC3) inhibitors as anticancer agents: A review. Eur J Med Chem.

[19] Cao F, Zwinderman MRH, Dekker FJ (2018). The process and strategy for developing selective histone deacetylase 3 inhibitors. Molecules.

[20] Glass CK, Rosenfeld MG (2000). The coregulator exchange in transcriptional functions of nuclear receptors. Genes Dev.

[21] Perissi V, Jepsen K, Glass CK, Rosenfeld MG (2010). Deconstructing repression: evolving models of co-repressor action. Nat Rev Genet.

[22] Karagianni P, Wong J (2007). HDAC3: taking the SMRT-N-CoRrect road to repression. Oncogene.

[23] Yu J, Li Y, Ishizuka T, Guenther MG, Lazar MAA (2003). SANT motif in the SMRT corepressor interprets the histone code and promotes histone deacetylation. EMBO J.

[24] Guenther MG, Yu J, Kao GD, Yen TJ, Lazar MA (2002). Assembly of the SMRT-histone deacetylase 3 repression complex requires the TCP-1 ring complex. Genes Dev.

[25] Guo C, Gow C-H, Li Y, Gardner A, Khan S, Zhang J (2012). Regulated clearance of histone deacetylase 3 protects independent formation of nuclear receptor corepressor complexes. J Biol Chem.

[26] Lewandowski SL, Janardhan HP, Trivedi CM (2015). Histone deacetylase 3 coordinates deacetylase-independent epigenetic silencing of transforming growth factor-β1 (TGF-β1) to orchestrate second heart field development. J Biol Chem.

[27] Yin H, Kang Z, Zhang Y, Gong Y, Liu M, Xue Y (2021). HDAC3 controls male fertility through enzyme-independent transcriptional regulation at the meiotic exit of spermatogenesis. Nucleic Acids Res.

[28] Montgomery RL, Potthoff MJ, Haberland M, Qi X, Matsuzaki S, Humphries KM (2008). Maintenance of cardiac energy metabolism by histone deacetylase 3 in mice. J Clin Invest.

[29] Jang J, Song G, Pettit SM, Li Q, Song X, Cai C-L (2022). Epicardial HDAC3 promotes myocardial growth through a novel microRNA pathway. Circ Res.

[30] Norwood J, Franklin JM, Sharma D, D’Mello SR (2014). Histone deacetylase 3 is necessary for proper brain development. J Biol Chem.

[31] Wang Y, Frank DB, Morley MP, Zhou S, Wang X, Lu MM (2016). HDAC3-dependent epigenetic pathway controls lung alveolar epithelial cell remodeling and spreading via miR-17-92 and TGF-β signaling regulation. Dev Cell.

[32] Navabi N, Whitt J, Wu S-E, Woo V, Moncivaiz J, Jordan MB (2017). Epithelial histone deacetylase 3 instructs intestinal immunity by coordinating local lymphocyte activation. Cell Rep.

[33] Kim TH, Yoo J-Y, Choi K-C, Shin J-H, Leach RE, Fazleabas AT (2019). Loss of HDAC3 results in nonreceptive endometrium and female infertility. Sci Transl Med.

[34] Ferrari A, Longo R, Fiorino E, Silva R, Mitro N, Cermenati G (2017). HDAC3 is a molecular brake of the metabolic switch supporting white adipose tissue browning. Nat Commun.

[35] Emmett MJ, Lim H-W, Jager J, Richter HJ, Adlanmerini M, Peed LC (2017). Histone deacetylase 3 prepares brown adipose tissue for acute thermogenic challenge. Nature.

[36] Hong S, Zhou W, Fang B, Lu W, Loro E, Damle M (2017). Dissociation of muscle insulin sensitivity from exercise endurance in mice by HDAC3 depletion. Nat Med.

[37] Armour SM, Remsberg JR, Damle M, Sidoli S, Ho WY, Li Z (2017). An HDAC3-PROX1 corepressor module acts on HNF4α to control hepatic triglycerides. Nat Commun.

[38] Ji H, Zhou Y, Zhuang X, Zhu Y, Wu Z, Lu Y (2019). HDAC3 deficiency promotes liver cancer through a defect in H3K9ac/H3K9me3 transition. Cancer Res.

[39] Ning L, Rui X, Bo W, Qing G (2021). The critical roles of histone deacetylase 3 in the pathogenesis of solid organ injury. Cell Death Dis.

[40] Finkel T (2012). Signal transduction by mitochondrial oxidants. J Biol Chem.

[41] Sun Z, Singh N, Mullican SE, Everett LJ, Li L, Yuan L (2011). Diet-induced lethality due to deletion of the Hdac3 gene in heart and skeletal muscle. J Biol Chem.

[42] Vallée A, Lecarpentier Y (2018). Crosstalk between peroxisome proliferator-activated receptor gamma and the canonical WNT/β-catenin pathway in chronic inflammation and oxidative stress during carcinogenesis. Front Immunol.

[43] Wójtowicz S, Strosznajder AK, Jeżyna M, Strosznajder JB (2020). The novel role of PPAR alpha in the brain: promising target in therapy of Alzheimer’s disease and other neurodegenerative disorders. Neurochem Res.

[44] Suntar I, Sureda A, Belwal T, Sanches Silva A, Vacca RA, Tewari D (2020). Natural products, PGC-1α, and Duchenne muscular dystrophy. Acta Pharma Sin B.

[45] Zhang Q, Liu X-M, Hu Q, Liu Z-R, Liu Z-Y, Zhang H-G (2021). Dexmedetomidine inhibits mitochondria damage and apoptosis of enteric glial cells in experimental intestinal ischemia/reperfusion injury via SIRT3-dependent PINK1/HDAC3/p53 pathway. J Transl Med.

[46] Wu N, Yin L, Hanniman EA, Joshi S, Lazar MA (2009). Negative feedback maintenance of heme homeostasis by its receptor, Rev-erbα. Genes Dev.

[47] Corbet C, Pinto A, Martherus R, Santiago de Jesus JP, Polet F, Feron O (2016). Acidosis drives the reprogramming of fatty acid metabolism in cancer cells through changes in mitochondrial and histone acetylation. Cell Metab.

[48] Chi Z, Chen S, Xu T, Zhen W, Yu W, Jiang D (2020). Histone deacetylase 3 couples mitochondria to drive IL-1β-dependent inflammation by configuring fatty acid oxidation. Mol Cell.

[49] Dávalos-Salas M, Montgomery MK, Reehorst CM, Nightingale R, Ng I, Anderton H (2019). Deletion of intestinal Hdac3 remodels the lipidome of enterocytes and protects mice from diet-induced obesity. Nat Commun.

[50] Iershov A, Nemazanyy I, Alkhoury C, Girard M, Barth E, Cagnard N (2019). The class 3 PI3K coordinates autophagy and mitochondrial lipid catabolism by controlling nuclear receptor PPARα. Nat Commun.

[51] Hayes JD, Flanagan JU, Jowsey IR (2005). Glutathione transferases. Annu Rev Pharm Toxicol.

[52] Xiang X, Dong G, Zhu J, Zhang G, Dong Z (2022). Inhibition of HDAC3 protects against kidney cold storage/transplantation injury and allograft dysfunction. Clin Sci.

[53] Liu J, Li G, Wang X, Wang L, Zhao R, Wang J (2016). Droxinostat, a histone deacetylase inhibitor, induces apoptosis in hepatocellular carcinoma cell lines via activation of the mitochondrial pathway and downregulation of FLIP. Transl Oncol.

[54] Zhu H, Shan L, Schiller PW, Mai A, Peng T (2010). Histone deacetylase-3 activation promotes tumor necrosis factor-α (TNF-α) expression in cardiomyocytes during lipopolysaccharide stimulation. J Biol Chem.

[55] Rinnerthaler M, Bischof J, Streubel MK, Trost A, Richter K (2015). Oxidative stress in aging human skin. Biomolecules.

[56] Zeeshan HMA, Lee GH, Kim H-R, Chae H-J (2016). Endoplasmic reticulum stress and associated ROS. Int J Mol Sci.

[57] Tu BP, Weissman JS (2004). Oxidative protein folding in eukaryotes: mechanisms and consequences. J Cell Biol.

[58] Wei H, Ma W, Lu X, Liu H, Lin K, Wang Y (2021). KDELR2 promotes breast cancer proliferation via HDAC3‐mediated cell cycle progression. Cancer Commun.

[59] Dahlby T, Simon C, Backe MB, Dahllöf MS, Holson E, Wagner BK (2020). Enhancer of zeste homolog 2 (EZH2) mediates glucolipotoxicity-induced apoptosis in β-cells. Int J Mol Sci.

[60] Plaisance V, Rolland L, Gmyr V, Annicotte J-S, Kerr-Conte J, Pattou F (2014). The class I histone deacetylase inhibitor MS-275 prevents pancreatic beta cell death induced by palmitate. J Diabetes Res.

[61] Chen M, Liu Q, Chen L, Zhang L, Gu E (2017). Remifentanil postconditioning ameliorates histone H3 acetylation modification in H9c2 cardiomyoblasts after hypoxia/reoxygenation via attenuating endoplasmic reticulum stress. Apoptosis.

[62] Schröder K (2020). NADPH oxidases: current aspects and tools. Redox Biol.

[63] Zhang Y, Murugesan P, Huang K, Cai H (2020). NADPH oxidases and oxidase crosstalk in cardiovascular diseases: novel therapeutic targets. Nat Rev Cardiol.

[64] Siuda D, Zechner U, El Hajj N, Prawitt D, Langer D, Xia N (2012). Transcriptional regulation of Nox4 by histone deacetylases in human endothelial cells. Basic Res Cardiol.

[65] Kong G, Huang Z, Ji W, Wang X, Liu J, Wu X (2017). The ketone metabolite β-hydroxybutyrate attenuates oxidative stress in spinal cord injury by suppression of class I histone deacetylases. J Neurotrauma.

[66] Rochette L, Lorin J, Zeller M, Guilland J-C, Lorgis L, Cottin Y (2013). Nitric oxide synthase inhibition and oxidative stress in cardiovascular diseases: Possible therapeutic targets?. Pharmacol Ther.

[67] Tejero J, Shiva S, Gladwin MT (2019). Sources of vascular nitric oxide and reactive oxygen species and their regulation. Physiol Rev.

[68] Förstermann U, Sessa WC (2012). Nitric oxide synthases: regulation and function. Eur Heart J.

[69] Li H, Förstermann U (2000). Nitric oxide in the pathogenesis of vascular disease. J Pathol.

[70] Zampetaki A, Zeng L, Margariti A, Xiao Q, Li H, Zhang Z (2010). Histone deacetylase 3 is critical in endothelial survival and atherosclerosis development in response to disturbed flow. Circulation.

[71] Jung S-B, Kim C-S, Naqvi A, Yamamori T, Mattagajasingh I, Hoffman TA (2010). Histone deacetylase-3 antagonizes aspirin-stimulated endothelial nitric oxide production by reversing aspirin-induced lysine acetylation of endothelial nitric oxide synthase. Circ Res.

[72] Zhang M-X, Zhang C, Shen YH, Wang J, Li X-N, Chen L (2008). Effect of 27nt small RNA on endothelial nitric-oxide synthase expression. Mol Biol Cell.

[73] Choi W-S, Seo Y-B, Shin P-G, Kim W-Y, Lee SY, Choi Y-J (2015). Veratric acid inhibits iNOS expression through the regulation of PI3K activation and histone acetylation in LPS-stimulated RAW264.7 cells. Int J Mol Med.

[74] Bagheri F, Khori V, Alizadeh AM, Khalighfard S, Khodayari S, Khodayari H (2016). Reactive oxygen species-mediated cardiac-reperfusion injury: mechanisms and therapies. Life Sci.

[75] Kukreja RC, Kontos HA, Hess ML, Ellis EF (1986). PGH synthase and lipoxygenase generate superoxide in the presence of NADH or NADPH. Circ Res.

[76] Chen X, Barozzi I, Termanini A, Prosperini E, Recchiuti A, Dalli J (2012). Requirement for the histone deacetylase Hdac3 for the inflammatory gene expression program in macrophages. Proc Natl Acad Sci USA.

[77] Peulen O, Gonzalez A, Peixoto P, Turtoi A, Mottet D, Delvenne P (2013). The anti-tumor effect of HDAC inhibition in a human pancreas cancer model is significantly improved by the simultaneous inhibition of cyclooxygenase 2. PLoS ONE.

[78] Kwon Y, Kim Y, Eom S, Kim M, Park D, Kim H (2015). MicroRNA-26a/-26b-COX-2-MIP-2 loop regulates allergic inflammation and allergic inflammation-promoted enhanced tumorigenic and metastatic potential of cancer cells. J Biol Chem.

[79] Wang Y, Branicky R, Noë A, Hekimi S (2018). Superoxide dismutases: dual roles in controlling ROS damage and regulating ROS signaling. J Cell Biol.

[80] Förstermann U, Xia N, Li H (2017). Roles of vascular oxidative stress and nitric oxide in the pathogenesis of atherosclerosis. Circ Res.

[81] Nozik-Grayck E, Woods C, Stearman RS, Venkataraman S, Ferguson BS, Swain K (2016). Histone deacetylation contributes to low extracellular superoxide dismutase expression in human idiopathic pulmonary arterial hypertension. Am J Physiol-Lung Cell Mol Physiol.

[82] Wen Q, Lau N, Weng H, Ye P, Du S, Li C, et al. Chrysophanol exerts anti-inflammatory activity by targeting histone deacetylase 3 through the high mobility group protein 1-nuclear transcription factor-kappa B signaling pathway in vivo and in vitro. Front Bioeng Biotechnol. 2021;8. https://www.frontiersin.org/article/10.3389/fbioe.2020.623866. 10.3389/fbioe.2020.623866 PMC7868569 33569375

[83] Liu X, Jiang C, Liu G, Wang P, Shi M, Yang M (2020). Sodium butyrate protects against oxidative stress in human nucleus pulposus cells via elevating PPARγ-regulated Klotho expression. Int Immunopharmacol.

[84] Rojo de la Vega M, Chapman E, Zhang DD (2018). NRF2 and the hallmarks of cancer. Cancer Cell.

[85] Hayes JD, Dinkova-Kostova AT (2014). The Nrf2 regulatory network provides an interface between redox and intermediary metabolism. Trends Biochem Sci.

[86] Chen F, Gao Q, Zhang L, Ding Y, Wang H, Cao W (2021). Inhibiting HDAC3 (histone deacetylase 3) aberration and the resultant Nrf2 (nuclear factor erythroid-derived 2-related factor-2) repression mitigates pulmonary fibrosis. Hypertension.

[87] Liu G-H, Qu J, Shen X (2008). NF-kappaB/p65 antagonizes Nrf2-ARE pathway by depriving CBP from Nrf2 and facilitating recruitment of HDAC3 to MafK. Biochim Biophys Acta.

[88] Guo X, Hong S, He H, Zeng Y, Chen Y, Mo X (2020). NFκB promotes oxidative stress-induced necrosis and ischemia/reperfusion injury by inhibiting Nrf2-ARE pathway. Free Radic Biol Med.

[89] Zhao Q, Zhang F, Yu Z, Guo S, Liu N, Jiang Y (2019). HDAC3 inhibition prevents blood-brain barrier permeability through Nrf2 activation in type 2 diabetes male mice. J Neuroinflammation.

[90] Martin D, Li Y, Yang J, Wang G, Margariti A, Jiang Z (2014). Unspliced X-box-binding protein 1 (XBP1) protects endothelial cells from oxidative stress through interaction with histone deacetylase 3. J Biol Chem.

[91] Vallabhapurapu S, Karin M (2009). Regulation and function of NF-kappaB transcription factors in the immune system. Annu Rev Immunol.

[92] Hayden MS, Ghosh S (2008). Shared principles in NF-kappaB signaling. Cell.

[93] Morgan MJ, Liu Z (2011). Crosstalk of reactive oxygen species and NF-κB signaling. Cell Res.

[94] Ea C-K, Baltimore D (2009). Regulation of NF-kappaB activity through lysine monomethylation of p65. Proc Natl Acad Sci USA.

[95] Saccani S, Marazzi I, Beg AA, Natoli G (2004). Degradation of promoter-bound p65/RelA is essential for the prompt termination of the nuclear factor kappaB response. J Exp Med.

[96] Chen L, Mu Y, Greene WC (2002). Acetylation of RelA at discrete sites regulates distinct nuclear functions of NF-kappaB. EMBO J.

[97] Wang D, Westerheide SD, Hanson JL, Baldwin AS (2000). Tumor necrosis factor alpha-induced phosphorylation of RelA/p65 on Ser529 is controlled by casein kinase II. J Biol Chem.

[98] Zhang F, Qi L, Feng Q, Zhang B, Li X, Liu C (2021). HIPK2 phosphorylates HDAC3 for NF-κB acetylation to ameliorate colitis-associated colorectal carcinoma and sepsis. Proc Natl Acad Sci USA.

[99] Leus NGJ, van der Wouden PE, van den Bosch T, Hooghiemstra WTR, Ourailidou ME, Kistemaker LEM (2016). HDAC 3-selective inhibitor RGFP966 demonstrates anti-inflammatory properties in RAW 264.7 macrophages and mouse precision-cut lung slices by attenuating NF-κB p65 transcriptional activity. Biochem Pharm.

[100] Shen Y, Yang R, Zhao J, Chen M, Chen S, Ji B (2022). The histone deacetylase inhibitor belinostat ameliorates experimental autoimmune encephalomyelitis in mice by inhibiting TLR2/MyD88 and HDAC3/ NF-κB p65-mediated neuroinflammation. Pharmacol Res.

[101] Molstad DHH, Mattson AM, Begun DL, Westendorf JJ, Bradley EW (2020). Hdac3 regulates bone modeling by suppressing osteoclast responsiveness to RANKL. J Biol Chem.

[102] Oh SK, Kim D, Kim K, Boo K, Yu YS, Kim IS (2019). RORα is crucial for attenuated inflammatory response to maintain intestinal homeostasis. Proc Natl Acad Sci USA.

[103] Liao Y, Cheng J, Kong X, Li S, Li X, Zhang M (2020). HDAC3 inhibition ameliorates ischemia/reperfusion-induced brain injury by regulating the microglial cGAS-STING pathway. Theranostics.

[104] Thatikonda S, Pooladanda V, Sigalapalli DK, Godugu C (2020). Piperlongumine regulates epigenetic modulation and alleviates psoriasis-like skin inflammation via inhibition of hyperproliferation and inflammation. Cell Death Dis.

[105] Kaestner KH, Knochel W, Martinez DE (2000). Unified nomenclature for the winged helix/forkhead transcription factors. Genes Dev.

[106] Gui T. Burgering BMT FOXOs: masters of the equilibrium. FEBS J. n/a. 10.1111/febs.16221. 10.1111/febs.16221 PMC10078705 34610198

[107] Klotz L-O, Sánchez-Ramos C, Prieto-Arroyo I, Urbánek P, Steinbrenner H, Monsalve M (2015). Redox regulation of FoxO transcription factors. Redox Biol.

[108] Olmos Y, Sánchez-Gómez FJ, Wild B, García-Quintans N, Cabezudo S, Lamas S (2013). SirT1 regulation of antioxidant genes is dependent on the formation of a FoxO3a/PGC-1α complex. Antioxid Redox Signal.

[109] Calnan DR, Brunet A (2008). The FoxO code. Oncogene.

[110] Nott A, Cheng J, Gao F, Lin Y-T, Gjoneska E, Ko T (2016). Histone deacetylase 3 associates with MeCP2 to regulate FOXO and social behavior. Nat Neurosci.

[111] Mihaylova MM, Vasquez DS, Ravnskjaer K, Denechaud P-D, Yu RT, Alvarez JG (2011). Class IIa histone deacetylases are hormone-activated regulators of FOXO and mammalian glucose homeostasis. Cell.

[112] Brunet A, Sweeney LB, Sturgill JF, Chua KF, Greer PL, Lin Y (2004). Stress-dependent regulation of FOXO transcription factors by the SIRT1 deacetylase. Science.

[113] Storz P (2011). Forkhead homeobox type O transcription factors in the responses to oxidative stress. Antioxid Redox Signal.

[114] Sack MN, Fyhrquist FY, Saijonmaa OJ, Fuster V, Kovacic JC (2017). Basic biology of oxidative stress and the cardiovascular system: part 1 of a 3-part series. J Am Coll Cardiol.

[115] Bai Y, Wang X, Zhao S, Ma C, Cui J, Zheng Y (2015). Sulforaphane protects against cardiovascular disease via Nrf2 activation. Oxid Med Cell Longev.

[116] Borrelli MA, Turnquist HR, Little SR (2021). Biologics and their delivery systems: trends in myocardial infarction. Adv Drug Deliv Rev.

[117] Su Q, Liu Y, Lv X-W, Dai R-X, Yang X-H, Kong B-H (2020). LncRNA TUG1 mediates ischemic myocardial injury by targeting miR-132-3p/HDAC3 axis. Am J Physiol Heart Circ Physiol.

[118] Liem DA, Zhao P, Angelis E, Chan SS, Zhang J, Wang G (2008). Cyclin-dependent kinase 2 signaling regulates myocardial ischemia/reperfusion injury. J Mol Cell Cardiol.

[119] Song K, Li L, Quan Q, Wei Y, Hu S (2020). Inhibited histone deacetylase 3 ameliorates myocardial ischemia-reperfusion injury in a rat model by elevating microRNA-19a-3p and reducing cyclin-dependent kinase 2. IUBMB Life.

[120] Mia MM, Cibi DM, Ghani SABA, Song W, Tee N, Ghosh S (2020). YAP/TAZ deficiency reprograms macrophage phenotype and improves infarct healing and cardiac function after myocardial infarction. PLOS Biol.

[121] Kao Y-H, Liou J-P, Chung C-C, Lien G-S, Kuo C-C, Chen S-A (2013). Histone deacetylase inhibition improved cardiac functions with direct antifibrotic activity in heart failure. Int J Cardiol.

[122] Wang Y-Y, Gao B, Yang Y, Jia S-B, Ma X-P, Zhang M-H (2022). Histone deacetylase 3 suppresses the expression of SHP-1 via deacetylation of DNMT1 to promote heart failure. Life Sci.

[123] Sharifi-Sanjani M, Shoushtari AH, Quiroz M, Baust J, Sestito SF, Mosher M (2014). Cardiac CD47 drives left ventricular heart failure through Ca2+-CaMKII-regulated induction of HDAC3. J Am Heart Assoc.

[124] Na J, Jin H, Wang X, Huang K, Sun S, Li Q (2021). The crosstalk of HDAC3, microRNA-18a and ADRB3 in the progression of heart failure. Cell Biosci.

[125] Li B, Yu Y, Liu K, Zhang Y, Geng Q, Zhang F (2021). β-Hydroxybutyrate inhibits histone deacetylase 3 to promote claudin-5 generation and attenuate cardiac microvascular hyperpermeability in diabetes. Diabetologia.

[126] Poleshko A, Shah PP, Gupta M, Babu A, Morley M, Manderfield LJ (2017). Genome-nuclear lamina interactions regulate cardiac stem cell lineage restriction. Cell.

[127] Zeisberg M, Neilson EG (2010). Mechanisms of tubulointerstitial fibrosis. J Am Soc Nephrol.

[128] Black LM, Lever JM, Agarwal A (2019). Renal inflammation and fibrosis: a double-edged sword. J Histochem Cytochem.

[129] Lin W, Zhang Q, Liu L, Yin S, Liu Z, Cao W (2017). Klotho restoration via acetylation of Peroxisome Proliferation-Activated Receptor γ reduces the progression of chronic kidney disease. Kidney Int.

[130] Chen F, Gao Q, Wei A, Chen X, Shi Y, Wang H (2021). Histone deacetylase 3 aberration inhibits Klotho transcription and promotes renal fibrosis. Cell Death Differ.

[131] Frederick JP, Liberati NT, Waddell DS, Shi Y, Wang X-F (2004). Transforming growth factor beta-mediated transcriptional repression of c-myc is dependent on direct binding of Smad3 to a novel repressive Smad binding element. Mol Cell Biol.

[132] Xi Q, Wang Z, Zaromytidou A-I, Zhang XH-F, Chow-Tsang L-F, Liu JX (2011). A poised chromatin platform for TGF-β access to master regulators. Cell.

[133] Liu L, Lin W, Zhang Q, Cao W, Liu Z (2016). TGF-β induces miR-30d down-regulation and podocyte injury through Smad2/3 and HDAC3-associated transcriptional repression. J Mol Med.

[134] Noh H, Oh EY, Seo JY, Yu MR, Kim YO, Ha H (2009). Histone deacetylase-2 is a key regulator of diabetes- and transforming growth factor-beta1-induced renal injury. Am J Physiol Ren Physiol.

[135] Wang X, Liu J, Zhen J, Zhang C, Wan Q, Liu G (2014). Histone deacetylase 4 selectively contributes to podocyte injury in diabetic nephropathy. Kidney Int.

[136] Levine MH, Wang Z, Bhatti TR, Wang Y, Aufhauser DD, McNeal S (2015). Class-specific histone/protein deacetylase inhibition protects against renal ischemia reperfusion injury and fibrosis formation. Am J Transpl.

[137] Gitler AD, Dhillon P, Shorter J (2017). Neurodegenerative disease: models, mechanisms, and a new hope. Dis Model Mech.

[138] Steffan JS, Bodai L, Pallos J, Poelman M, McCampbell A, Apostol BL (2001). Histone deacetylase inhibitors arrest polyglutamine-dependent neurodegeneration in Drosophila. Nature.

[139] Bates EA, Victor M, Jones AK, Shi Y, Hart AC (2006). Differential contributions of *Caenorhabditis elegans* histone deacetylases to huntingtin polyglutamine toxicity. J Neurosci.

[140] Janczura KJ, Volmar C-H, Sartor GC, Rao SJ, Ricciardi NR, Lambert G (2018). Inhibition of HDAC3 reverses Alzheimer’s disease-related pathologies in vitro and in the 3xTg-AD mouse model. Proc Natl Acad Sci USA.

[141] Han KA, Shin WH, Jung S, Seol W, Seo H, Ko C (2017). Leucine-rich repeat kinase 2 exacerbates neuronal cytotoxicity through phosphorylation of histone deacetylase 3 and histone deacetylation. Hum Mol Genet.

[142] Shan B, Xu C, Zhang Y, Xu T, Gottesfeld JM, Yates JR (2014). Quantitative proteomic analysis identifies targets and pathways of a 2-aminobenzamide HDAC inhibitor in Friedreich’s ataxia patient iPSC-derived neural stem cells. J Proteome Res.

[143] Matheson R, Chida K, Lu H, Clendaniel V, Fisher M, Thomas A (2020). Neuroprotective effects of selective inhibition of histone deacetylase 3 in experimental stroke. Transl Stroke Res.

[144] Kuboyama T, Wahane S, Huang Y, Zhou X, Wong JK, Koemeter-Cox A (2017). HDAC3 inhibition ameliorates spinal cord injury by immunomodulation. Sci Rep.

[145] Selkoe DJ, Hardy J (2016). The amyloid hypothesis of Alzheimer’s disease at 25 years. EMBO Mol Med.

[146] Zhu X, Wang S, Yu L, Jin J, Ye X, Liu Y (2017). HDAC3 negatively regulates spatial memory in a mouse model of Alzheimer’s disease. Aging Cell.

[147] Shukla S, Tekwani BL (2020). Histone deacetylases inhibitors in neurodegenerative diseases, neuroprotection and neuronal differentiation. Front Pharm.

[148] Lee H-Z, Kwitkowski VE, Del Valle PL, Ricci MS, Saber H, Habtemariam BA (2015). FDA approval: belinostat for the treatment of patients with relapsed or refractory peripheral T-cell lymphoma. Clin Cancer Res.

[149] Mann BS, Johnson JR, Cohen MH, Justice R, Pazdur R (2007). FDA approval summary: vorinostat for treatment of advanced primary cutaneous T-cell lymphoma. Oncologist.

[150] Dolskiy AA, Pustylnyak VO, Yarushkin AA, Lemskaya NA, Yudkin DV (2017). Inhibitors of histone deacetylases are weak activators of the FMR1 gene in fragile X syndrome cell lines. Biomed Res Int.

[151] Raedler LA (2016). Farydak (Panobinostat): first HDAC inhibitor approved for patients with relapsed multiple myeloma. Am Health Drug Benefits.

[152] Rai M, Soragni E, Chou CJ, Barnes G, Jones S, Rusche JR (2010). Two new pimelic diphenylamide HDAC inhibitors induce sustained frataxin upregulation in cells from Friedreich’s ataxia patients and in a mouse model. PLoS ONE.

[153] Chou CJ, Herman D, Gottesfeld JM (2008). Pimelic diphenylamide 106 is a slow, tight-binding inhibitor of class I histone deacetylases. J Biol Chem.

[154] Wagner FF, Lundh M, Kaya T, McCarren P, Zhang Y-L, Chattopadhyay S (2016). An isochemogenic set of inhibitors to define the therapeutic potential of histone deacetylases in β-cell protection. ACS Chem Biol.

[155] Terracciano S, Di Micco S, Bifulco G, Gallinari P, Riccio R, Bruno I (2010). Synthesis and biological activity of cyclotetrapeptide analogues of the natural HDAC inhibitor FR235222. Bioorg Med Chem.

[156] Wang C, Henkes LM, Doughty LB, He M, Wang D, Meyer-Almes F-J (2011). Thailandepsins: bacterial products with potent histone deacetylase inhibitory activities and broad-spectrum antiproliferative activities. J Nat Prod.

[157] Zhang L, Chen Y, Jiang Q, Song W, Zhang L (2019). Therapeutic potential of selective histone deacetylase 3 inhibition. Eur J Med Chem.

